# Transactional sex work and HIV among women in conflict-affected Northeastern Uganda: a population-based study

**DOI:** 10.1186/s13031-022-00441-5

**Published:** 2022-02-25

**Authors:** Jennifer J. Mootz, Omolola A. Odejimi, Aishwarya Bhattacharya, Bianca Kann, Julia Ettelbrick, Milena Mello, Milton L. Wainberg, Kaveh Khoshnood

**Affiliations:** 1 Department of Psychiatry, Columbia University, 1051 Riverside Drive, New York, NY 10032 USA; 2 New York State Psychiatric Institute, 1051 Riverside Drive, Kolb 171, New York, NY 10032 USA; 3 Educational Psychology, Texas Tech University, 2500 Broadway, Lubbock, TX 79409 USA; 4 Department of Psychology, Yale University, New Haven, CT 06520 USA; 5 The New School, Eugene Lang College, 72 5th Avenue, New York, NY 10011 USA; 6 School of Public Health, Yale University, 60 College St, New Haven, CT 06510 USA

**Keywords:** War, Sex work, HIV, Uganda, Low-income country, Sub-Saharan Africa

## Abstract

**Background:**

Armed conflict and the HIV pandemic are significant global health issues. Evidence of the association between armed conflict and HIV infection has been conflicting. Our objective was to examine the role of mediating risk factors, such as engagement in transactional sex work, to elucidate the relation between armed conflict and HIV infection.

**Methods:**

We used multistage sampling across three Northeastern Ugandan districts to randomly select 605 women aged 13 to 49 to answer cross-sectional surveys from January to May of 2016. We used multivariate logistic regression model with R 4.0.3 to examine if exposure to armed conflict has an indirect effect on reporting having an HIV-positive serostatus through engagement in transactional sex work. Age and district residence were included as covariates.

**Results:**

Exposure to armed conflict *β* = .16, *SE* = .04, *p* < .05, *OR* = 1.17, 95% [CI .08, .23] was significantly associated with reporting a HIV-positive serostatus. For each 1-unit increase in exposure to armed conflict (i.e., additional type of armed conflict exposure), there was a 17% increase in the odds of reporting a HIV-positive serostatus. Engagement in transactional sex work was not associated with reporting a HIV-positive serostatus *β* = .04, *SE* = .05, *p* = .37, 95% [CI − .051, .138]. We found district of residence, age, and interaction effects.

**Conclusions:**

Although exposure to armed was associated with reporting an HIV-positive serostatus, this relationship was not mediated by engagement in transactional sex. Further research is needed on risk factors that mediate this relationship. The likelihood of reporting a HIV-positive serostatus increased with each additional type of exposure to armed conflict. Thus, screening for exposure to multiple traumatic stressors should occur in HIV prevention settings. Healthcare services that are trauma-informed and consider mental distress would likely improve HIV outcomes.

## Background

Over 1.5 billion people from 40 countries live in areas considered to be impacted by active armed conflict. Armed conflict can result in large-scale population displacement across borders [1, 2], and lead to violations of human rights [3, 4], deleterious public health circumstances [5, 6], weakened health systems [7, 8] and breakdown of traditional social structures [9]. Armed conflict is also associated with socio-economic devastation due to destruction of institutions and human capital as well as disruption of commerce [10], agriculture and education [11]. Due to its negative impact on population health and health systems, armed conflict has been described as a significant public health issue [12].

While a few published studies have identified armed conflict as a key contributor to the global HIV epidemic, evidence has been sparse and conflicting [13, 14]. Civil wars have been consistently correlated with an increase in the loss of disability-adjusted life years due to HIV infection [15, 16]. Conflict-related increase in prevalence of HIV infection prevalence has been theorized to occur through a number of mechanisms. Violent conflict leads to the deployment of soldiers as well as displacement of civilians which can increase contact between populations [16]. Soldiers and insurgents have exhibited a higher prevalence of HIV infection compared to civilians [17, 18] and their mobility can increase chances for the spread of HIV [6]. Additionally, armed conflict displaces the local population creating a group of asylum seekers that are vulnerable to HIV infection [19, 20]. Refugees are exposed to conditions of poverty, lack of access to healthcare, and greater incidence of sexual violence which increases their risk of acquiring HIV/AIDS [21, 22]. For instance, 60–80% of survivors of rape during the Rwandan genocide were estimated to be seropositive for HIV compared to 13.5% of the general population [4, 23, 24].

Other studies have provided contrasting evidence. One meta-analysis from 2007 found no relation between HIV prevalence and conflict across seven Southern African countries [25]. Others have found either no association between conflict and HIV prevalence [26] or insufficient evidence for the same [27]. This has led to an understanding of conflict as a potential risk factor for increased incidence for HIV infections, whose effect may be mediated by other factors in the causal pathway. While a global analysis found no direct association between HIV disability adjusted life years (DALYs) and armed conflict among WHO member states, this study demonstrated that risk factors such as illicit drug use and increased alcohol consumption moderated the conflict-HIV infection pathway [13]. This analysis also showed that country level indicators, such as number of displaced people, amount of HIV spending, ethnic heterogeneity (increasing possibility of political contention and disadvantaged groups or populations), and number of people on antiretroviral therapy mediated the pathway between conflict and HIV DALYs. However, more theoretical models are needed [28].

Transactional sex work (TSW) has been proposed as one such mediator of the relationship between conflict-related trauma and HIV infection in post-conflict settings [29]. TSW refers to non-commercial sexual relationships with a component of intimacy, where sex is exchanged for material gain [30]. This informal exchange of transactional sex can occur for cash, food, clothing, transport, items for family, school fees, housing and/or for the advancement of social status [31–34]. There are three dominant paradigms when considering TSW and its relation to HIV: deprivation (meeting basic needs), agency (improving social status), and instrumentality (receiving material goods as expressions of love). Much of the literature about TSW as a mediator of HIV infection in armed conflict settings highlights TSW as a means to meet basic needs due to economic deprivation and extreme poverty. The forced migration and population displacement that result from armed conflict can break down family structures and affect women’s livelihood and economic security [35]. Due to conflict-induced economic instability and food scarcity, women engage in TSW to avoid starvation and family destitution [36, 37]. For example, in Northern Uganda, women turned to TSW as a survival strategy when farming was no longer feasible [38].

Additionally, differential power dynamics have been shown to be associated with engaging in TSW. For example, refugee camps are traditionally controlled primarily by male committees that can abuse power by demanding sex in exchange for distribution of food and resources [39]. Peacekeepers, stationed in humanitarian settings, have also been reported to engage in TSW with vulnerable refugees [40, 41]. Such transactions tend to occur between younger women and older men which contributes to a power asymmetry that leaves women less able to negotiate condom use or prevent coercion [42].

TSW makes women vulnerable to HIV infection [43], other sexually transmitted infections (STIs), unwanted pregnancy, and intimate partner violence [44]. Women engaging in TSW with two or more casual partners or with a partner who has more than a five-year age difference have been found to be at a higher risk of HIV infections [33, 45]. Young women are particularly susceptible to adverse health outcomes in post-conflict settings due to weakening of healthcare infrastructure and resources that support reproductive health, as well as breakdown of law and order and community services [35, 46, 47]. For example, 80% of healthcare workers fled or were killed during the Rwandan genocide, and hospital equipment was looted or destroyed [48]. While a growing body of literature has speculated that TSW is responsible for the disproportionately high HIV prevalence amongst young women compared to young men even in peacetime settings [49, 50], there are exceptions that highlight the complexity of this relation. One study, for example, found that among urban refugees and displaced adolescents and young adults in Kampala, Uganda, TSW associated with higher condom use and self-efficacy among adolescent girls and women. Although, this relation was not replicated among boys and men engaged in TSW demonstrating a gendered component to this association.

This study aimed to qualify the mediating effect of TSW on the observed relationship between exposure to armed conflict and reporting a HIV-positive serostatus in three districts across Northeastern Uganda. We hypothesized that armed conflict would associate with reporting a HIV-positive serostatus and that TSW would indirectly mediate this association.

## Methods

### Study setting

This study is part of a larger study that tested pathways between armed conflict and intimate partner violence and mental health outcomes of interpersonal violence (authors blinded; authors blinded). In the present study, we examined an indirect pathway mediated by transactional sex work between women’s exposure to armed conflict and reporting a HIV-positive serostatus in rural communities within the Katakwi, Amuria, and Kumi districts of Teso subregion in Northeastern Uganda. HIV prevalence among adults aged 15–24 in rural Uganda is 5.8% (6.7% females 4.7%, males 4.7%) [51]. Media reports suggest that the prevalence of HIV infection in Katakwi increased from 9 to 21% by the end of 2005 [52] and anecdotal evidence put the prevalence rate at 17% [29]. Teso has around 1.8 million residents and is one of the least urbanized and poorest regions of Uganda [53]. Around 10% of the population has never attended school and only about 6% have completed secondary education. Iteso women have a lower literacy rate than men (64% women; 84% men) [54] and less access to resources. Only 15% of women compared to 33% of men have their own mobile phones, and 5% of women have had access to life time use of the internet compared to 20% of men [53]. Around 13.7% of young people in Uganda are estimated to engage in sex work [55], and the estimated prevalence of HIV infection among sex workers is 33% [56].

In Teso, conflict has occurred in the form of violent looting of cattle and livestock by the Karamojong, a nomadic, pastoral ethnic group, that have been conducting cattle raids within sub-tribes since the 1940s. However, the level of armed conflict in the region has exacerbated after the Karamojong acquired thousands of AK-47 s abandoned by Idi Armin’s soldiers after Armin was ousted by the Tanzanian army [57]. This conflict has led to large scale population displacement into protected camps within Teso [58]. In 2003, the Lord’s Resistance Army invaded Teso and committed mass murders, rape and looting that led to civilian displacement, the burning of homes, and the abduction of children [59]. Although the Ugandan government started a disarmament program in Karamoja, several communities have remained militarized with the presence of government soldiers who protect against reoccurring cattle raiding [60].

### Study design

After obtaining ethics approval from institutional review boards in Uganda and the US, our local collaborator identified six local research team members from Northeastern Uganda who were fluent in Ateso and English. The lead author, a licensed psychologist, provided a 3-day training on interviewing strategies of asking about sensitive and stigmatized issues, such as exposure to armed conflict, HIV testing and status, and TSW, with an affirming and nonjudgmental approach. Training also addressed safety concerns and provided an overview of the study protocol. The team reviewed and modified the instruments that two independent translation professionals had forward and back translated after facilitating a small pilot of the survey administration.

Using a multistage sampling strategy in collaboration with our local partners who had anecdotal data on severity of exposure to armed conflict based on their programming activities, we selected three districts in Teso: Katakwi District (high exposure), Amuria District (medium exposure), and Kumi District (low exposure). Eight villages were randomly selected from subcounties within each district thought to characterize the district’s exposure level. From January to May of 2016, in three teams of pairs, the research team spun a pen to sample every third household in the center of each village. Eligible participants included all women aged 18 to 49 and married (self-defined by participants and including legal or customary) girls under age 18. A minimum of 25 participants was surveyed in each village (*n* = 605). The response rate was 87% (67 households had no eligible participants; 4 households did not speak the local language; and 20 eligible women declined participation). Research team members randomly selected one participant from eligible household members. After securing a private location, the research team members obtained verbal consent because of low literacy. Participants received resources and a bar of soap (recommended by our local partners) upon survey completion.

### Instruments

We measured self-reported exposure to armed conflict using an adapted *Exposure to Political Violence Inventory* (Clark et al., 2010). Six items inquired about various forms of respondent exposures (yes/no), such as physical, verbal, sexual, relocation, abduction, and lost work. We constructed a sum score of exposure events from this checklist and measured it continuously. The internal consistency (Cronbach’s alpha) for the six exposure to armed conflict items was 0.96.

We used a shortened *Survey of Women’s Health and Life Experiences in Uganda: Women’s Survey* (World Health Organization, 2005) to measure self-reported demographic and HIV status (positive, negative, or unknown). We first asked participants about whether they have received HIV testing (yes/no) and then asked about the results. To represent income, we constructed a sumscore of material possessions that included ownership of home electricity, radio, tv, phone, refrigerator, bicycle, motorcycle, car, solar panel and land (yes/no).

The TSW variable was measured with a question from the *International Men and Gender Equality Survey: Women’s Questionnaire* [48], asking whether women have sex with a male partner for the following reasons: *Provided you with food, clothes, cell phone or transportation; Paid you school fees or residence fees; Provided you with somewhere to stay; Gave you cosmetics or money for beauty products; Gave items for you children or family; Gave you cash or money to pay your bills; Provided you anything else that you could not afford by yourself.* We created a sum score of TSW from this checklist. The internal consistency of the sum score was 0.98.

### Data analysis

Missingness was assumed to be missing at random in which the reason for missingness was not associated with any variables in the dataset. Under this assumption, missing data were handled using the Full Information Maximum Likelihood estimation which uses the available information to inform the estimated parameters and their standard errors in the presence of missing information [61]. No cases were excluded from the analysis.

We used R version 4.0.3 to summarize demographics, frequencies, and run model analyses. A two-way contingency table was created to look at the association between living in the three districts and HIV status. To calculate measures of association, odds ratios were calculated from the contingency table. Odds ratios described the odds of positive HIV status given living in one of the three districts in the study.

A multivariate logistic regression model was used to test the hypothesized model that there is an indirect pathway from exposure to armed conflict and HIV status through engagement in TSW. Logistic regression was used because the outcome of interest, HIV status, was a binary response. Predictors and interaction terms were then added to the model. To find the best model by comparing different sets of predictors, nested model comparisons using the chi squared difference test were done. Bootstrap estimates of the standard errors were created to construct confidence intervals for parameter estimates in the logistic regression model.

Three models were estimated using the dummy coded technique. Model 1 contained predictors representing residence in district 1 and 2, model 2 contained district 1 and 3, and model 3 contained district 2 and 3. The group not included in the model is used as the reference category in model interpretation. Income, age, the interaction of income and district, the interaction of district and age, and the interaction of income and age were all used as covariates and pruned if not significant. Main effects were left in the model for significant interactions. The effects of these covariates differed depending on which district was the reference group. Odds ratios are reported for estimates of the influence of a main effect on the outcome and not reported for interaction terms or non-significant results.

## Results

### Participants

The sample consisted of women and married adolescent girls whose ages ranged from 13 to 49 years *(M* = 29.88). The sample was further described by HIV status in Table 1. Most of the respondents (92.8%) were partnered during the survey and almost all (98.6%) had been partnered at least once in their lifetime. Women, on average, had four children with ages ranging from 0 to 13 years. Literacy was low with 89 women (15%) having received no education and 363 (60%) reporting they were unable to read or write. Women were primarily Anglican (46%) and Catholic (45.6%). The most common forms of exposure to armed conflict were verbal harassment (~ 60%) and relocation (~ 60%). Approximately 21% of women reported engaging in TSW and 7.6% reported having a HIV-positive serostatus. While the entire sample consisted of 605 participants, we retained only those individuals who had been tested for HIV (n = 593).

**Table 1:** Participant Demographics by HIV Testing Status

Variables	Tested		Untested	
	HIV positive (n = 46)	HIV Status HIV negative (n = 541)	Unknown (n = 12)	All (n = 605)
*Age (years)*				
M (SD)	35.91 (7.67)	29.37 (8.73)	29.78 (12.14)	29.88 (8.89)
Range	19–48	13–49	18–48	13–49
Literacy (% literate)	53.3	67.1	33.3	39.70
*Number of children*				
M (SD)	5.46 (2.49)	4.20 (2.90)	4.33 (3.20)	4.51 (3.14)
Range	1–10	0–15	1–10	0–13
*Armed conflict exposure n (%)*				
Verbal	38 (82.60)	315 (59.50)	9 (81.10)	367 (60.70)
Physical	6 (13.04)	28 (5.30)	1 (9.10)	35 (5.80)
Sexual	1 (2.20)	4 (0.75)	1 (9.10)	5 (0.82)
Relocated	37 (82.20)	310 (58.70)	8 (72.70)	360 (59.50)
Abduction	7 (15.60)	47 (8.90)	0 (0.00)	54 (8.90)
TSW n (%) Yes	10 (21.74)	112 (20.70)	4 (33.33)	127 (21.00)

Not all respondents who tested for HIV reported their status

*TSW* transactional sex work

While there were some missing observations, nonresponse was low. Variables in the model ranged in missingness from 0% to 3.13%. In the chi square test of indepence, results indicated that districts did not significantly differ from each other in incidence of HIV, *X*^2^ = 4.19, df = 2, *p* = 0.12. After comparing odds ratios, confidence intervals contained 1, indicating the odds of HIV status were not different for districts. Although these tests of independence and odds ratios indicated there was not a greater chance of having HIV in one district over the other, these tests do not consider any other variables in the model, which can impact associations among variables. The multivariate logistic regression model allowed further exploration of this relationship in the presence of other variables.

### Armed conflict, TSW, and HIV

In the initial model, the mediated pathway was tested and no covariates were included. Exposure to armed conflict (EAC), *β* = 0.16, *SE* = 0 0.04, *p* < 0.05, *OR* = 1.17, 95% [CI 1.09, 1.27], was strongly associated with HIV status.The estimated odds ratio that a respondent had a reported a HIV-positive serostatus for each unit increase in EAC was 1.17. For each 1-unit increase in EAC (i.e., additional type of armed conflict exposure), there was a 17% increase in the odds of having a reporting a HIV-positive serostatus. Engagement in TSW was not associated with reporting a HIV-positive serostatus, *β* = 0.04, *SE* = 0.05, *p* = 0.37. The bootstrap confidence intervals derived from 1000 samples indicated that the indirect effect (cross-sectional mediation) coefficient was not significant, *β* = −0.002, *SE* = 0.00, *p* = 0.25, which did not support the hypothesis that the relation between exposure to armed conflict and HIV status has a significant indirect pathway through engagement in TSW.

### Model 1 (reference Group District 3)

**Fig. 1:**
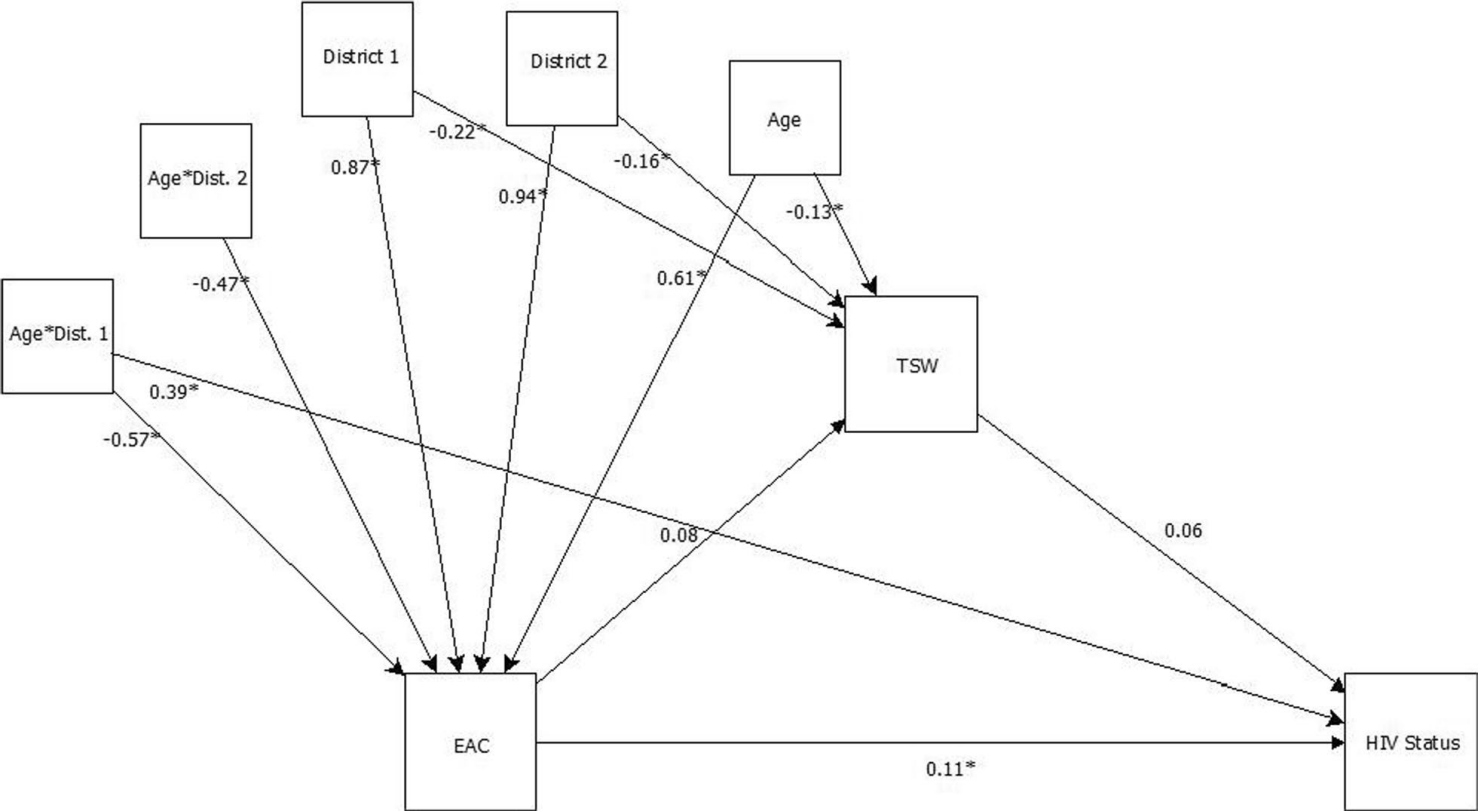
Model for reference group district 3. *EAC* Exposure to Armed Conflict, *TSW* Transactional Sex Work; *significant at .05 level

In this analysis, all estimates regarding district can be compared to District 3. Respondent EAC was marginally associated with reporting a HIV-positive serostatus, *β* = 0.11, *SE* = 0.05, *p* = 0.05, *OR* = 1.12, 95% [CI 1.01, 1.23]. For every unit increase in EAC, there was a 12% increase in the odds of a reported HIV-positive serostatus. Figure 1 is a graphical representation of Model 1.

The interaction of age and living in District 1 was associated with reporting a HIV-positive serostatus, *β* = 0.38, *SE* = 0 0.19, *p* < 0.05, *OR* = 1.46, 95% [CI 1.01, 2.12]. For respondents living in District 1, for each 1-year increase in age, there was a 46% increase in the odds ofreporting a HIV-positive serostatus, when compared to respondents in District 3. Older women in District 1 were more likely to have reported a HIV-positive serostatus when compared older women in District 3. The interaction of age and district was associated with EAC. The interaction of age and living in District 1 had a negative relationship with EAC, *β* = −0.57, *SE* = 0.14, *p* < 0 0.05, *OR* = 0.57 95% [CI 0.43, 0.74]. EAC also had a negative relationship with the interaction of age and living in District 2, *β* = −0.47, *SE* = 0 0.16, *p* < 0.05, *OR* = 0.63, 95% [CI 0.46, 0.86]. Compared to younger respondents in District 3, younger respondents living in Districts 1 and 2 are exposed to higher levels of EAC. Living in Districts 1 and 2 had a strong association with respondents’ engagement in TSW. Those in District 1, *β* = −0.27, *SE* = 0.05, *p* < 0.05, *OR* = 0.76, 95% [CI 0.69, 0.84], reported 0.76 times the odds of engaging in TSW than women living in District 3. Those in District 2, *β* = −0.16, *SE* = 0.06, *p* < 0.05, *OR* = 0.85, 95% [CI 0.76, 0.96], reported 0.85 times the odds engaging in TSW than women living in District 3. Age also had a strong association with engagement in TSW, *β* = −0.13, *SE* = 0.04, *p* < 0.05, *OR* = 0.88, 95% [CI 0.81, 0.95] indicating that younger respondents reported 0.88 times the odds of engaging TSW than older women in District 3.

### Model 2 (reference Group District 2)

All estimates regarding district can be compared to District 2 in this model. Respondent EAC was associated with reporting a HIV-positive serostatus, *β* = 0.09, *SE* = 0.00, *p* < 0.05, *OR* = 1.09, 95% [CI 1.09, 1.10]. For every unit increase in EAC, respondents had a 0.09 times the odds to have reported a HIV-positive serostatus. Age had a positive association with reporting a HIV-positive serostatus, *β* = 0.17, *SE* = 0.04, *p* < 0.05, *OR* = 1.19, 95% [CI 1.10, 1.28]. For each 1-year increase in age, there was a 19% increase in the odds of having reported a HIV-positive serostatus. Older women more likely to report a HIV-positive serostatus in District 2. The interaction of age and living in District 3 was strongly associated with EAC. The interaction of age and living in District 3 had a positive relationship with EAC, *β* = 0.52, *SE* = 0.13, *p* < 0.05, *OR* = 1.68, 95% [CI 0.30, 2.17]. When compared to older respondents living in District 3, older respondents in District 2 were exposed to higher levels of armed conflict. Age also had a significant association with engagement in TSW, *β* = −0.10, *SE* = 0.05, *p* < 0.05, *OR* = 0.90, 95% [CI 0.82, 1.00] indicating that younger respondents reported 0.90 times the odds of engaging in TSW than older women.

### Model 3 (reference Group District 1)

All estimates regarding district can be compared to District 1 in this analysis. Respondent EAC was associated with reporting a HIV-positive serostatus, *β* = 0.10, *SE* = 0.05, *p* < 0.05, *OR* = 1.11, 95% [CI 1.00, 1.22]. For every unit increase in EAC, there wasa11% increase in the odds of having reported a HIV-positive serostatus. The interaction of age and living in District 3 had a strong association with HIV status, *β* = −0.38, *SE* = 0.19, *p* < 0.05, *OR* = 0.68, 95% [CI 0.47, 0.99]. For respondents living in District 3, for each 1-year decrease in age, there were 0.68 times the odds in having reported a HIV-positive serostatus, when compared to respondents in District 1. Younger women in District 3 were less likely to have reported a HIV-positive serostatus, when compared younger women in District 1. This finding coincides with the partitioning of residuals for the test for independence in the contingency table (Table 2). That test found marginal significance in the test of independence of reporting a HIV-positive serostatus and living in either District 1 or 3. Those results indicated a possible nonindependence of the observed association where differences may lie in the grouping of the categories. The interaction of age and living in District 3 had a strong association with EAC, *β* = 0.56, *SE* = 0.14, *p* < 0.05, *OR* = 1.75, 95% [CI 1.33, 2.30]. For those living in District 3, each 1-year increase in age related to higher levels of armed conflict exposure. Older women living in District 3 had a greater association with EAC, when compared to older women living in District 1. Age also had an association with engagement in TSW, *β* = −0.10, *SE* = 0.05, *p* < 0.05, *OR* = 0.90, 95% [CI 0.82, 1.00] indicating that younger respondents reported 0.90 times the odds of engaging in TSW than older women.

**Table 2:** Multivariate Regression Model Estimates for Model 1 (Reference Group—District 3)

Regression	*Β*	*SE*	z	*p* value
TSW on EAC	0.08	0.05	1.42	0.16
HIVstat on TSW	0.06	0.05	1.31	0.19
HIVstat on EAC	0.11	0.05	1.94	0.05
*HIVstat on*				
District 1	− 0.42	0.12	− 2.09	0.04
Age:Dist1	0.39	0.19	2.05	< 0.05
*EAC on*				
Age	0.61	0.06	10.30	< 0.05
District 1	0.87	0.13	6.91	< 0.05
District 2	0.94	0.14	6.57	< 0.05
Age:Dist1	− 0.57	0.14	− 4.12	< 0.05
Age:Dist2	− 0.47	0.16	− 3.00	< 0.05
*TSW*				
Age	− 0.13	0.04	− 3.01	< 0.05
District 1	− 0.22	0.05	− 4.47	< 0.05
Income:Dist2	− 0.19	0.05	− 3.61	< 0.05

*SE* standard error, *TSW* transactional sex work, *EAC* exposure to armed conflict

## Discussion

To our knowledge, this is one of the first population-based studies from sub-Saharan Africa to examine transactional sex work (TSW) as a mediator in an indirect pathway between exposure to armed conflict and reporting a HIV-positive serostatus. While our study showed that reporting a HIV-positive serostatus was associated with exposure to armed conflict, TSW did not mediate this pathway. Age and residence, however, were associated with armed conflict and reporting a HIV-positive serostatus.

Consistent with previous research on the link between exposure to armed conflict and HIV [6, 13, 62], we found that exposure to armed conflict was strongly associated with reporting a HIV-positive serostatus. Additionally, there was an interaction between age and HIV infection such that older women residing in the high conflict district were more likely to have reported a HIV-positive serostatus than women residing in districts with less exposure to conflict. The nature of the conflict in the highly exposed district likely has caused major breakdowns in healthcare and education infrastructure because it has transpired for decades. A UNAIDS report on security in humanitarian settings noted that the relation between armed conflict and HIV infection could be mediated by the interruption of social networks, economic vulnerability of women and children, and disruption of health services [63]. Participants from the high exposure district reported the highest prevalence of HIV (around 11%) as compared to an approximate HIV prevalence of 6% in the lesser-exposed districts. More research is needed to examine the association between environmental variables, such as disruption to infrastructure, and HIV infection.

Notably, the likelihood of reporting a HIV-positive serostatus increased by 9–17% with each additional type of exposure to armed conflict. A study in the US found that respondents living with HIV who had multiple prior traumatic exposures were more likely to experience being diagnosed with HIV as traumatic and exhibited lower quality of life than those with fewer exposures [64]. Enduring multiple traumatic events has demonstrated a cumulative effect on the presence and severity of mental distress and health problems [65, 66]. Individuals living with HIV and comorbid mental distress have demonstrated poorer adherence to treatment in a meta-analysis of 95 studies [67], and shown to be less likely to achieve viral suppression [68]. Despite the numerous challenges to accessing health and mental healthcare in conflict-affected settings, especially for sex workers [69], our findings suggest that HIV screening and prevention in healthcare settings should include exposure to traumatic events and mental health sequelae, particularly in populations that have endured armed conflict. A review of trauma-informed interventions for HIV prevention and treatment found only eight studies globally, five of which were from LMICs. Most studies intervened with women and girls and no studies addressed exposure to armed conflict. Thus, future programming should consider remedying gaps in the treatment of men and including community-level exposure to violence [70]. Additionally, having multiple traumatic exposures may warrant more urgency when triaging patients for specialized mental health care. For providers not trained in evidence-based mental health interventions, adopting a trauma-informed approach to care could be helpful. This would include creating a trauma-sensitive environment, screening for trauma, educating patients on the effects of trauma on health and behavior, and providing referrals for specialized providers when possible [71].

Contrary to previous findings [33, 44], our study found that TSW did not have an indirect effect on the relation between armed conflict and reporting a HIV-positive serostatus. This finding may have occurred due to the fact that we did not collect data on frequency and duration of TSW [72], knowledge of paying partner’s HIV status, or age difference between partners [42, 73, 74]. In addition to TSW, other determinants of HIV prevalence in armed conflict populations may include minimal access to contraceptive options, such as condoms, and violence against women [75]. A study that examined the effects of mass rape in conflict-affected areas and the possibility of this mediating HIV infection found that mass rape could contribute to a 6–7% median annual increase in infections due to above average HIV rates among soldiers and the efficiency of HIV transmission during violent sex [76]. Additionally, a systematic review of the literature on the relation between TSW and HIV infection noted that a range of measures were used to operationalize TSW [30], and these measurements often conflated TSW with sex work (a sexual exchange for money or goods that often lacks intimacy) [50]. A standardization or streamlining of the measurement system would be beneficial in designing more general interventions that can be more widely applicable. Further research is necessary to continue examining the mechanisms through which HIV infection is related to armed conflict.

The findings of this study should be considered within its limitations. First, the data were cross-sectional preventing conclusions about causality. Next, we measured TSW by occurrence of exchanging transactional sex for various goods. Perhaps assessing behaviors such as contraceptive use while engaging in TSW would have better represented how TSW might be related to armed conflict and HIV status. Similarly, we dichotomized HIV status and potentially would have observed different results if other sexually transmitted illnesses had been included. Finally, assessing stigmatized economic activities and health conditions such as TSW and HIV status through self-report methods via face-to-face interviews could have resulted in underreporting due to social desirability bias and lack of status awareness.

## Conclusion

We conducted a population-based study with women living in three districts in Northeastern Uganda with varying levels of exposure to armed conflict. While transactional sex work did not mediate a pathway between exposure to armed conflict and reporting a HIV-positive serostatus, each additional exposure to armed conflict increased the likelihood of women reporting a HIV-positive serostatus by 9–17%. Standardization of transactional sex work measurement may help clarify its relation to armed conflict and HIV status. Healthcare services that focus on HIV prevention and treatment that are trauma-informed and consider mental health would likely improve outcomes.

## Data Availability

The datasets used and/or analysed during the current study are available from the corresponding author on reasonable request.
